# Research on factors affecting tourist involvement in coffee tourism after the COVID-19 pandemic in Thailand

**DOI:** 10.12688/f1000research.123759.1

**Published:** 2022-11-08

**Authors:** Warach Madhyamapurush

**Affiliations:** 1 Lecturer of Tourism and Hotel management, School of Business and Communication Art, University of Phayao, Phayao Province, Thailand

**Keywords:** Coffee Tourism, COVID-19 pandemic, Foreign Tourists, Tourist Behaviors, Stochastic Neuro-Fuzzy Decision Tree (SNF-DT).

## Abstract

**Background:** The world economy was broken by the COVID-19 pandemic, which affected the coffee industry. The COVID-19 pandemic's financial effects might influence equity markets and personal lives. This includes financial commodities like coffee, which the pandemic is predicted to damage. Coffee tourism is an emerging new kind of tourism in Thailand, formed in response to growing demand from visitors with a particular affinity for the beverage. Coffee tourism may contribute considerably to the expansion of Thai tourism if given the proper guidance and assistance.

**Methods:** As part of a coffee tourism experience focusing on first-hand activities and information, tourists can visit neighbouring sites while on a coffee plantation. This research uses a stochastic neuro-fuzzy decision tree (SNF-DT) to analyse coffee tourism in Thailand. The research surveys 400 international and Thai coffee tourists. According to studies, Thai visitors mostly visit coffee tourism locations in Thailand for enjoyment. They also wanted to visit coffee fields in order to get personal knowledge of coffee production and marketing. Based on the comments of Thai visitors, coffee tourism in northern Thailand looks to be highly and effectively handled. Due to the same factor, responses from foreign coffee tourists indicated that many of their journeys to coffee tourism destinations were made entirely for enjoyment rather than the business. They also wanted to meet local tour guides and acquire handmade and locally produced things to understand more about coffee tourism.

**Result:** According to study results, coffee tourism management in northern Thailand looks well-received by international tourists. We also compare the suggested model to the traditional one to demonstrate its efficacy. The performance metrics are prediction rate, prediction error, and accuracy. The estimated results for our proposed technique are prediction rate (95%), prediction error (97%), and accuracy (94%).

## Introduction

**Figure 1.**
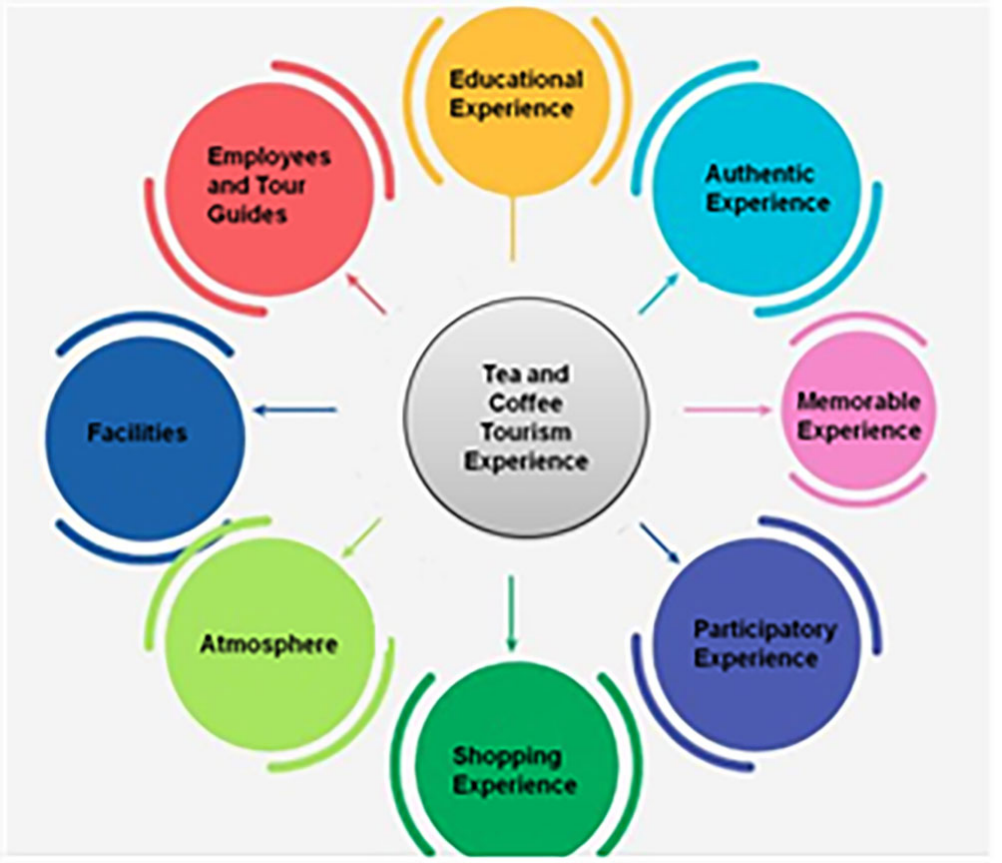
Factors of tourism experience (source: author).

One of Thailand’s key economic sectors, tourism, has consistently shown promise and development throughout time. Many travelers are looking to satisfy their need for a coffee-related experience to satisfy their taste for coffee. Such visitor demands provide Thailand the chance to distinguish its tourism goods via a distinctive brand identity, promote its cultures and distinctive characteristics, and work towards achieving global recognition. This coffee tour should let travelers appreciate coffee tourism. Tourists enjoy fresh coffee from its origin, the hilly environment, coffee farms, harvesting operations, bean roasting, etc. Tourism might become a national brand. The COVID-19 epidemic has had a significant impact on many areas of society. As the COVID-19 pandemic spreads, the financial impact on commodities markets and human lives is expected to be enormous and far-reaching. As a result of the pandemic, cash crops like coffee are also projected to be adversely impacted. Farmers in over 52 nations depend on coffee to make a living. ^1^ The epidemic also impacts cafes and restaurants that get their ingredients from these industries since they must adhere to rigorous food and hygiene regulations. Cafes and restaurants must follow current standards and newly updated COVID-19 health guidelines even though there have been no reported incidents of the virus being transmitted via food in any nation. ^2^ Coffee shops, among other food and beverage businesses, feel the effects of the new regulations, which many economists and analysts say are already severe. Major global businesses like tourism have been harmed by COVID-19’s unprecedented effects. ^3^ Figure 1 depicts factors of tourism experience.

Tourism and hospitality enterprises benefit from the coffee industry in this region. Risk perception and health guidelines have considerably impacted this, resulting in a decrease in coffee foot traffic and an increase in at-home usage. Furthermore, this pandemic has already had a severe impact on the worldwide coffee supply chain, which has resulted in a changing price for the beverage. ^4^ There is little doubt that coffee is one of the most important cash crops in the world today. A claim has been made that coffee tourism is part of the culinary travel industry. The variety of cultural experiences, distinct coffee-related customs, and unique coffee processing facilities in each location fueled brand growth for the destinations. For coffee tourists, collecting and amassing coffee-related experiences is one of the main attractions. ^5^ The potential of coffee tourism for sustainable livelihood and conservation is examined by Sustainable livelihood in southwest Ethiopia. The mixed-methods approach used the memorable tourism experiences scale (MTES) to analyze the tourism experiences offered by coffees in Taiwan. More qualitative research on coffee tourism is required. ^6^^,^^31^ A case study method is used to examine how COVID-19 affects the coffee industry and the accompanying consequences for tourism, as well as crisis management techniques for a post-pandemic environment in Taiwan. This study’s inability to completely implement the framework of the strategic approach is one of its weaknesses. ^7^^,^^32^ Qualitative research was not efficient for the analysis of coffee tourism. An empirical study to determine the role of coffee tourism in livelihood based on real-time data is not performed To overcome this issue, we introduce a machine learning approach in this study.

### Related works

As stated in Ref. 6, the coffee tourism system in every tourist spot must be continually improved, and measures should be taken to share information about coffee growth in Northern Thailand’s tourism sustainability.

A comprehensive literature review technique for tea and coffee tourism has been proposed in Ref. 7, which aims to identify the opportunities and obstacles of expanding such niche products for locations. This study’s methodology uses an English-language literature review of the previous studies on tea and coffee tourism. 31 papers on coffee tourism and 33 papers regarding tea tourism have been examined in total. Many studies have used a single stakeholder’s viewpoint or the viewpoint of a particular coffee/tea tourism setting, like buyers’ or manufacturers’, according to the findings of this investigation. Because this research is the first of its kind, it showed potential suggestions for future work in coffee and tea tourism.

Community-based tourism (CBT) is a growing topic of study, and this study ^8^ provides a framework to understand it grounded in experimentally verified socio-psychological, phenomenological, and cybernetic conceptions of behaviour. One of the most significant outcomes of this study is a more structured knowledge of how socio-psychological variables might support and enable a successful CBT business plan to achieve a desirable “sustainable” goal.

Analyzing the characteristics that influence the success of coffee agro-tourism and also designing an efficient mechanism for community-based coffee tourism have been the goals of this research. A total of 142 people from the Sukaratu District agrotourism sector took part in. ^9^ Coffee agrotourism throughout the Galunggung tourism region was shown to be sustained by the coffee plantation, according to the study findings.

Using data gleaned from a number of researches on the Thai coffee market, the author of this thesis makes recommendations for resolving the market’s current issues. According to the limited capacities of Ref. 10, inadequate data and analysis of information, the facts and actual conditions could vary, therefore more complete inquiries and analyses of the current system and problems are required, so as to put up more effective remedies and proposals. Although the Thai coffee industry has been thoroughly examined, there are still issues that need to be addressed and solutions found.

Depending upon that extended TPB framework, ^11^ intends to analyze the factors. Using the TPB structure as a starting point, this study provides a preliminary examination of the elements that may influence Thai consumers’ purchase intentions for certified coffee. According to the findings, self-identification has a greater impact on attitude than social identity, whereas attitude has the greatest impact on consumer buying behavior consequently.

Based on an extensive theoretical map that shows how an unfunded community member had carefully conceptualized a system of actions and endeavors that have a significant impact on society, ^12^ provides a practical example of a conceptually rich strategic vision. It is feasible and increased by attempts to expand common values with buyers, communities, and other relevant parties to establish sustainability-oriented principles.

Another study ^13^ found that customer happiness and consumer loyalty are directly linked to service quality. As a result, cost fairness and consumer loyalty are influenced positively by a company’s ability to keep its customers happy. Additionally, client satisfaction serves as a bridge connecting service quality and satisfaction. In addition, perceived price can operate as a bridge between a company’s ability to satisfy its customers and maintain their loyalty.

This research investigated the drivers of Norwegian senior tourists’ desire to visit Thailand and analyzed a path model of variables impacting their travel intention throughout the pre-visit stage. This research hypothesized that travel motivation, expectation, limitations, destination image, and electronics word of mouth (e-WOM) impact travel intention. The authors also explored participants’ travel sentiment about Thailand. Analysis was done on 500 samples of eligible responders. Using Linear structural relations (Lisrel) software version of 8.72, a structural equation model (SEM) was run to evaluate the factors affecting tourism. The impact investigation was carried out to see how study constructs had an impact. About 62% of such variation in tourist perception is explained by its predictors. ^14^

It was the focus of Ref. 15 to examine the possibility and possibilities of coffee tourism throughout Ethiopia’s south-western highlands. Here, we are looking at how sustainable tourism may support rural community livelihoods while also helping to protect biodiversity, particularly the wild Arabica coffee gene pool, in the montane forest. According to the findings, a fragile livelihood strategy and a desire to increase revenue are to blame for expanding agriculture into the forest and destroying the unique wild Arabic coffee gene pool.

There has been a rise in the popularity of agritourism in developing nations, which has been under-researched by academics. ^16^ They are helping to further the conversation about the advantages and disadvantages of coffee tourism by conducting a study based on the perspectives of many stakeholders. According to the study, farmers reap the most benefits from empowering and cooperating, diversifying their businesses, and creating a more sustainable environment.

As per Ref. 17, coffee tourism can potentially improve locals’ lives. This is because there are new markets to enter and benefits to be gained. Local communities and the environment benefit from strong government responsibilities, which should be supported.

In Ref. 18, there are several ways in which innovation and coffee tourism are linked in Gangneung, South Korea. By using coffee tourism as an example of food and drink tourism, they examined how previous research has linked meals and drinks with creativity before explaining how tiny towns like Gangneung, South Korea, have been able to thrive as coffee tourism operators and how their use of creative thinking contributed to its success.

The Cibulao landscape/environment was positively impacted by tourism. ^19^ Strong interactions between actors, resources, and the local governing structure led to sound economic and social performance. Enhancing the economy’s performance is essential as a vital component of sustainable use of resources led by good local governance regulation. According to the study’s findings, Community-based Coffee Tourism (CbCT) is further expanded as a green tourism technique to help the community’s economy while also improving the environment.

The purpose of Ref. 20 is to discover the most important factors influencing people’s desire to visit sustainable coffee and tea estates. In addition, the study examined the impact of travelers’ fears about contracting COVID-19 on their decision to visit coffee and tea tourism hotspots. A survey of 302 eco-conscious Gen Y and Z customers was conducted online using the idea of planned behavior as a foundation. The data were analyzed using partial least squares. Buyers’ opinions regarding healthy coffee/tea tourism have been influenced by their desire to learn and unwind. Regarding sustainable coffee/tea tourism, risk and mindset are the most critical factors. Consumers’ attitudes and behaviors toward a rapidly expanding type of tourism, which is taking place amid historically unprecedented conditions, can now be better captured by the planned behavior by including contemporary factors.

The article ^21^ outlines Lviv’s coffee tourism growth. The intended market for coffee tourism has been determined. Characteristics and significant components of the resource basis for the growth and operation of coffee tourism in Lviv are described. The local community and business work together to build and promote specialized infrastructure, tourist products (goods and services), and coffee tourism events. It aims to establish a pleasant urban hospitality area for both locals and visitors and to exhibit Lviv’s 200-year-old multiethnic coffee culture and distinctive coffee urban environment to the globe. The article includes representative statistics, data on Lviv’s coffee tourist offer, and survey findings. ^22^

In the coffee business, a reengineering effort focuses on identifying current innovations, evaluating existing ones, and designing new ones that can be implemented in a contingency. Changes, production, and transmission of information necessitate structural reengineering. Depending on the number of assets and external pressures, this is a new bet that will be made to rebuild the coffee industry; new jobs must be created, including those of a leader, communicator, and strategist.

Researchers ^23^ are interested in learning more about how to boost revenue during the COVID-19outbreak era in Central Aceh Regency by increasing coffee tourism, both physically and non-physically, using existing local expertise as a basis. Researchers used a descriptive qualitative research design and gathered responses from 40 coffee shop owners and patrons in various tourist destinations. Results revealed that the Central Aceh Regency offers significant potential for developing a coffee tourism model depending on the local tradition and enhancing income, particularly for those actively engaged in ecotourism development and coffee growers.

### Problem statement

The issues faced by Thailand’s coffee farmers are explored in this research. Weather variations, rising labor rates, and an increase in plant diseases have contributed to several failures throughout the decades. Comparing the state of coffee tourist experiences from both the supply and demand sides was the primary focus of the investigation. Tourists are unpredictable. A data-organized questionnaire was circulated to the respondents to analyze coffee farmers’ concerns. Our proposed model can handle numerical and category data and manage issues with several outputs. As more data points are utilized for training the tree, it increases the forecast data exponentially while comparing with other existing approaches.

### Contributions of this paper

• Stop word removal, stemming, dimensionality reduction, Min-max normalization is used for data preprocessing.
• Linear Discriminant Analysis (LDA) is used for feature extraction.
• Bat Algorithm is used for feature selection.
• Stochastic Neuro Fuzzy Decision Tree (SNF-DT) is used for data analysis.

## Methods

**Figure 2.**
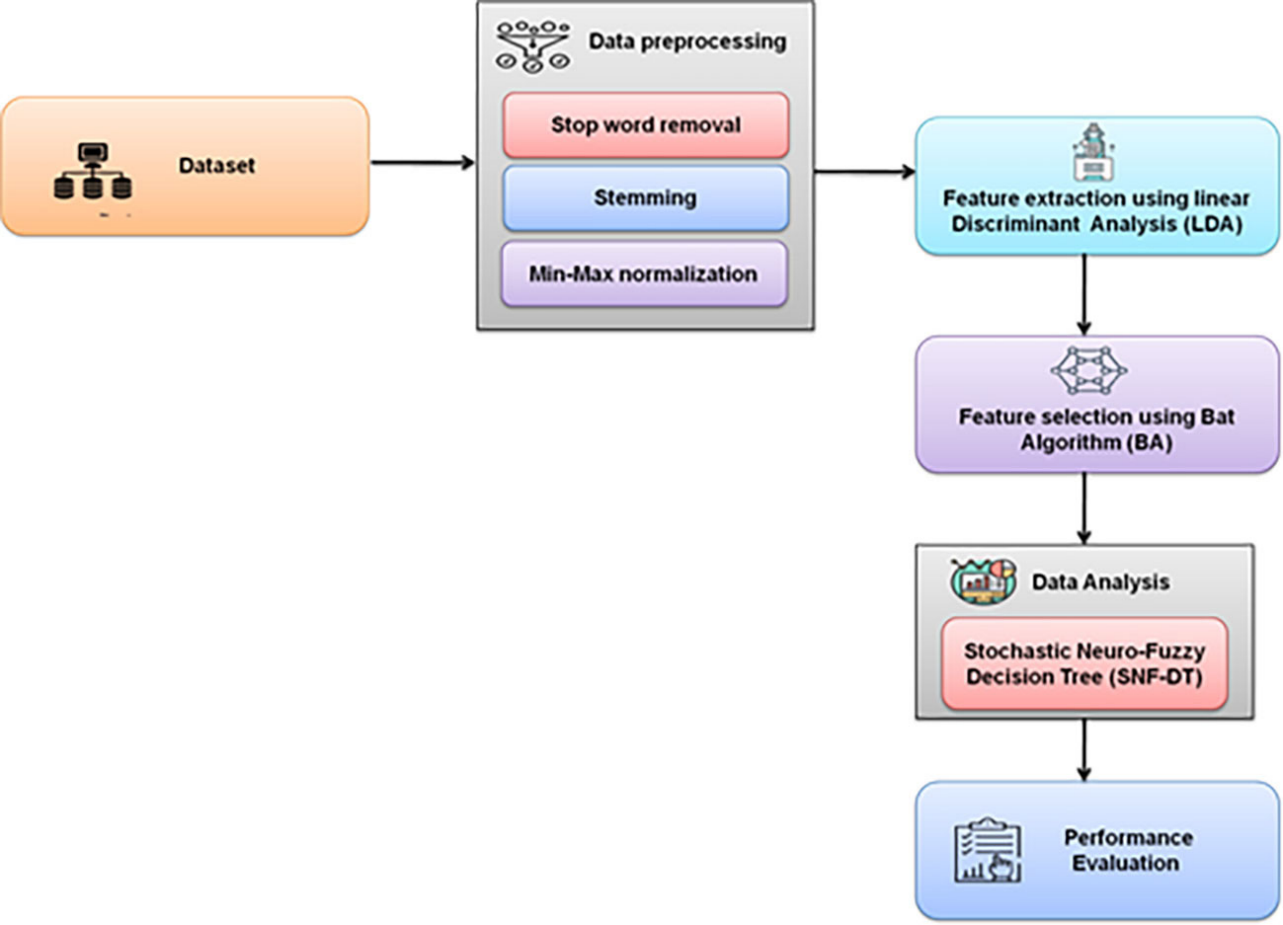
Schematic representation of the proposed methodology.

The COVID-19 pandemic might affect the coffee tourism industry in four ways: first, changes in demand in major markets, including short- and long-term changes. Secondly, effects on coffee production in the area from rules influencing production capacity, inputs, or employees. Third, hurdles and conflicts in getting coffee beans to markets such as transportation costs, border checks, etc. Furthermore, the crisis brought structural changes in the global economy, such as diminished accessibility to commerce. A quantitative method to investigate the effects of coffee tourism from COVID-19 was used for this research. The COVID-19 pandemic’s unexpected effect has influenced everyone in the coffee industry. Risks such as these are linked to adverse effects on health and mobility, and the economy. Hence deep analysis of the factors affecting coffee tourism after a pandemic is required. Figure 2 depicts the overall methodology used.

### Methodology

A survey was created to learn more about the behaviours, demands, and satisfaction levels of visitors who engaged in coffee tourism in the northern region of Thailand. Validity testing was performed on the completed questionnaires. Following that, the data were encoded and examined using statistical analysis tools. Stop word removal, stemming, dimensionality reduction, and min-max normalization are used for data preprocessing. Linear Discriminant Analysis (LDA) is used for feature extraction. Bat Algorithm is used for feature selection. Stochastic Neuro Fuzzy Decision Tree (SNF-DT) is used for data analysis.

### Ethics and consent

The Human Ethics Committee of the Faculty of Tourism and Faculty Management Program at University of Phayao gave its permission to the research protocol approval number 2.2/005/64; the date of approval was 03/23/2021. All participants also gave oral consent to take part in the study and received information about the research’s methodology. This method of consent was approved by the Human Ethics Committee.

### Data collection and processing

6,485,791 Thai and international visitors who traveled to the provinces of Chiang Rai, Chiang Mai, Mae Hong Son, and Lampang made up the population for this research. This study’s sample, chosen using accidental sampling and convenience sampling, consists of Thai and foreign tourists on vacation in coffee tourism destinations in Chiang Rai, Chiang Mai, Mae Hong Son, and Lampang provinces who participated in coffee-related tourism activities such as a coffee farm tour or coffee tasting. Yamane (1973) method was used to compute the sample size at a 95% confidence level with a 0.05 margin of error. ^24^^,^^34^

Although the result was 398, it was rounded up to 400 to make data gathering easier. Additionally, a percentage is used to show how many responders there were in each region.

Visitors from Thailand were asked to complete the questionnaire in Thai, while tourists from English-speaking countries were asked to complete the questionnaire in English. The questionnaire was in pen and paper format, and participants were required to complete the entire questionnaire. 2 participants who were approached to take part declined. Table 1 shows the sample groups in each province.

**Table 1.** Sample group in each province. ^25^

Province	Percentage	Number of tourists (person)	Sample group (persons)	Number of respondents (persons)	Sample group (persons)
Chiang Mai	38	Thai 2,463,541	151.93	152	
	25	Foreigner 1,624,755	100.20	100	
Lampang	5	Thai 312,534	19.28	19	
	1	Foreigner 33,189	2.05	2	
Chiang Rai	24	Thai 1,553,663	95.82	96	
	5	Foreigner 316,025	19.49	19	
Mae Hong Son	2	Thai 131,218	8.09	8	
	1	Foreigner 50,866		3	3.14

### Research instruments

A survey was created to learn more about the behaviors, demands, and satisfaction levels of visitors who engaged in coffee tourism in the northern region of Thailand. The survey consists of closed-ended questions about the respondent’s general characteristics, behaviors, needs, and satisfaction with the destination(s) for coffee tourism in terms of those destinations’ potential, with an emphasis on the attractions, accessibility, amenities, available package, activities, and ancillary services as defined by Buhalis’ 6As framework. The questionnaire included many options (based on a five-point rating scale) to help respondents adequately answer the closed-ended questions.

### Instrument validation

The questionnaire’s validity was thoroughly investigated. Each item on the questionnaire, primarily assessed by the thesis adviser, was scrutinized by five specialists. An Index of Item Objective Congruence (IOC) was used to measure the content validity and language suitability. The acceptable range is shown by the IOC score of 0.926. ^32^ After this, the questionnaire was modified following the expert’s advice, before being delivered to the thesis advisor for final revisions. A total of 30 visitors who were not part of the sample group completed the finished questionnaire as a test run. Cronbach’s alpha coefficient was used to gauge reliability. The output was 0.948, regarded as statistically valid. ^33^

### Procedures

The researcher used the validated questionnaire to gather data from the samples between April 2021 and March 2022. The information was gathered in several districts throughout the provinces of Chiang Rai, Chiang Mai, Mae Hong Son, and Lampang, where coffee tourism activities were planned.

### Data analysis

Validity testing was performed on the completed questionnaires. Following that, the data were encoded and examined using statistical analysis tools.

### Data preprocessing

Data preprocessing using stop word removal, stemming, term weighting, and dimension reduction are included.

### Stop word removal

In many natural language processing (NLP) applications, stop word removal is among the most often utilized test sets. The idea is to effectively remove words that exist in all of the corpus papers. Stop words frequently include adjectives and pronouns. Stop words are often eliminated from the text before deep learning and machine learning models are trained since stop words are plentiful and provide little to no unique information for classification or clustering. Stop words may help to reduce the size of your index and query by removing them. When it comes to performance, fewer words are usually better. Because stop words are semantically empty, they have no effect on relevance rankings for data preprocessing.

### Stemming

Stemming is the process of reducing words to their base by eliminating extraneous letters, generally a suffix, and removing inflection. Porter and Snowball are two examples of stemming models. The findings may be used to find similarities and links in massive datasets. Stemming is the text preparation normalization job associated with eliminating word affixes in natural language processing (prefixes and suffixes).

### Min-max normalization

The dataset was preprocessed using min-max normalization to give each element in the dataset a value ranging between 0 and 1. It establishes a data range by subtracting the greatest and lowest values of each data element. The least value of data elements is subtracted from actual data element in the numerator. The normalization of data is carried out according to equation 1.

$$x_{i}^{\prime }=\frac{x_{i}-x_{\min}}{x_{\max}-x_{\min}}$$ (1)

Where $x_{i}$ refers to the original value of input dataset, $x_{i}^{\prime }$ denotes the normalized value, $x_{\max}$ and $x_{\min}$ are the maximum and minimum values of the data respectively.

### Feature extraction using linear discriminant analysis (LDA)

Data transformation into numerical features that may be handled while keeping the information in the original data set is known as feature extraction. LDA is one of the most widely used linear algorithms for feature extraction. LDA is used to discover a lower-dimensional space that best distinguishes between data from various categories. The optimal way to separate data from various categories is to utilise a lower-dimensional space found by LDA. With this approach, the Fisher criteria, which is an objective function, is aimed towards maximisation:

$$Y\left(E\right)=\frac{\left|E^{P}D_{v}E\right|}{\left|E^{P}D_{e}E\right|}$$ (2)

$$\text{Where}\;D_{v}=\sum_{x=1}^{f}t_{x}\left(n_{x}-n\right){\left(n_{x}-n\right)}^{P},$$ (3)

De=∑x=1ftxWx∈fxi−nxi−nxP (4)

Each of these is referred to as an Inter- or Intraclass scattering matrix. $t_{x}=m_{x}/m$ is the prior probability of a sample belonging to class x with expectation W. E may be derived by solving $E^{*}$ = argmaxY(E) in solution space $L^{s\times t}=\left\{E\in Q^{s\times t},E^{P}E=X\right\}$ . This may be achieved by solving the following extended eigenvalues reduction issue: $D_{v}e=\lambda D_{e}e$ . The label information on the samples is used directly by LDA to solve classification issues.

### Feature selection using Bat algorithm

A predictive model’s input parameters are narrowed down via a process known as ‘feature selection’. BAT algorithm is used for feature selection.

### Bat algorithm ^26^

Researchers from different professions have been fascinated by bats, which have a unique capacity to echolocate. Bats, especially microbats, use echolocation as a kind of sonar: they generate a loud and brief pulse of sound, wait for it to strike an object, and then wait for the echo to return to their ears. In this way, bats may calculate their distance from an item. As a result of this remarkable orienting system, bats can distinguish a barrier from a prey item even in full darkness, enabling them to hunt. Bat algorithm is a novel meta-heuristic optimization approach based on observations of bat behaviour. To mimic the behaviour of a colony of bats that uses echolocation to locate prey and food, such a system has been devised. The following are some of the idealized principles used to represent this method:

• When it comes to detecting distance and distinguishing between food and background obstacles, bats employ echolocation in a remarkable manner.
• Searching for prey, an unidentified bat $t_{j}$ flew in random direction $y_{j}$ and velocity $w_{j}$ while emitting sound at the fixed frequency $f_{\min}$ and loudness of $B_{0}$ at the given location and time. They may automatically alter the wavelength λ (or frequency) of their produced pulses and the rate of pulse emission $s\in \left[0,1\right]$ , based on the closeness of their target.

While it’s possible for the loudness to fluctuate in numerous ways, imagine that the loudness ranges from a big (positive) $B_{0}$ to a minimal constant $B_{\min}$ .

The Bat algorithm is shown in Algorithm 1

**Algorithm 1: Bat algorithm.**

Objective purpose $f\left(y\right),y=\left(y^{1},\ldots ,y^{n}\right)$

Set up the bat population $y_{j}$ and $w_{j}$ , j=1,2,…,n.

Define the frequency of pulses $f_{j}$ at $y_{j}$ , ∀j=1,2,..,n.

Set up the pulse rates $s_{j}$ and the volume of sound $B_{j},j=1,2,\ldots ,n.$

While t<T

For every bat $t_{j}$ , do

Make novel solution by equation 5, 6, 7

If $\mathrm{rand}>s_{i}$ , then

Choose one of the finest options from the list.

Create a local solution based on the best one.

If $\mathrm{rand}<B_{j}$ and $f\left(y_{j}\right)<f\left(\hat{y}\right)$ , then

Explore novel solutions.

      Higher $s_{i}$ and lower $B_{j}$

  Sort the bats & discover recent finest $\hat{y}$ .

The movement of the digital bats is determined by updating their velocity and location using equations 5, 6, 7 for every time step u, where U is the maximum number of iterations are as follows:

$$f_{j}=f_{\min}+\left(f_{\min}-f_{\max}\right)\beta$$ (5)

$$w_{j}^{k}\left(u\right)=w_{j}^{k}\left(u-1\right)+\left[y^{k}-y_{j}^{k}\left(u-1\right)\right]f_{j}$$ (6)

$$y_{j}^{k}\left(u\right)=y_{j}^{k}\left(u-1\right)+w_{j}^{k}\left(u\right)$$ (7)

Beta $\left(\beta \right)$ - random integer created between the values zero and one.

$y_{j}^{k}\left(u\right)$ - at time step *u*, the value of the decision parameter *k* for the bat.

The results of $f_{j}$ are utilised to regulate the speed and range of bat movement. The variable ${\hat{y}}^{k}$ reflects the current global best position (solution) for the decision variable k, which is produced by comparing all of the *n* bats’ answers.

To increase the range of feasible options, random walks are introduced. Firstly, one of the finest solutions currently available is chosen, and every bat that accepts the criteria has its own solution generated using a random walk.

$$y_{\mathrm{new}}=y_{\mathrm{old}}+\in \bar{B}\left(u\right)$$ (8)

$\bar{B}\left(u\right)$ - overall bat noise level at *t*.

For each iteration of the algorithm, the loudness $B_{j}$ and the emission pulse rate $s_{j}$ are updated, as follows

Every iteration of the process, the $B_{j}$ and $s_{j}$ parameters are modified, as follows:

$$B_{j}\left(u+1\right)=\alpha B_{j}\left(u\right)$$ (9)

$$s_{j}\left(u+1\right)=s_{j}\left(0\right)\left[1-\exp\left(-\mathrm{\gamma u}\right)\right]$$ (10)

and α and γ -ad-hoc constants.

$s_{j}\left(0\right)$ and $B_{j}\left(0\right)$ are frequently chosen randomly.

In general, $B_{j}\left(0\right)\in \left[1,2\right]$ and $s_{j}\left(0\right)\in \left[0,1\right]$ .

### Data analysis using stochastic neuro fuzzy decision tree (SNF-DT)

Data analysis is the act of analysing, cleaning, manipulating, and modeling data in order to identify usable data, inform conclusion, and assist decision-making. Stochastic neuro fuzzy decision tree (SNF-DT) is used for data analysis.

### Stochastic neuro fuzzy decision tree (SNF-DT)

**Figure 3.**
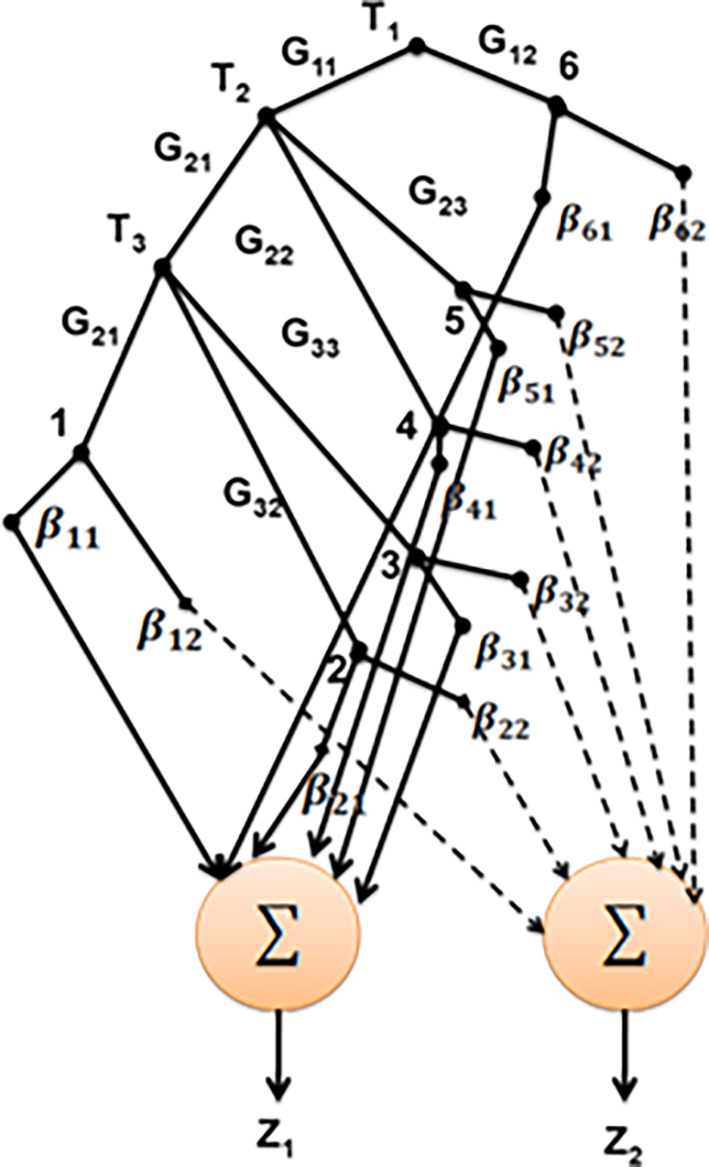
Structure of SNF-DT.

Incorporating neural learning algorithms into the feedback loop of hierarchical FDT (fuzzy decision tree) is the goal of stochastic neuro fuzzy decision tree (SNF-DT). The approach considerably increases the accuracy of classification of FDT without sacrificing the comprehensibility of the FDT structure. By returning from each leaf node to the root node, the back propagation learning method may be directly applied to the SNF-DT structure. A single forward cycle of FDT induction precedes numerous rounds of back propagation to fine-tune the FDT parameters in SNF-DT (membership functions and leaf certainties). Because of this approach, the hierarchical structure of the FDT tree isn’t disrupted, and the tree parameters may be tuned efficiently while still being interpretable. Figure 3 depicts the structure of SNF-DT.

Two summing nodes have been added to the basic SNF-DT structure in Figure 3 in order to perform inference. To determine $z_{1}$ , the certainty factors for class 1 (Yes) from each leaf node are added together. In the same approach, to determine $z_{2}$ ., the certainty factors corresponding to class 2 (No) are added together. To determine the strength of the mth class discharging at the nth leaf node in any pattern, use the formula

$$\mu_{\mathrm{pat}h_{n}}^{j}\times \beta_{\mathrm{nm}}$$ (11)

On the basis of the input characteristics accessible in traveling from the root node to the nth leaf node, each $p{\mathrm{ath}}_{n}\left(n=2,\ldots ,6\right)$ is created. $\mu_{\mathrm{pat}h_{n}}^{j}$ is the $\mathrm{pat}h_{n}$ membership degree. Which may be estimated as

$$\mu_{\mathrm{pat}h_{n}}^{j}=\prod_{k=1,2,3}\mu G_{\mathrm{kn}}\left(T_{k}^{j}\right)$$ (12)

The degree of confidence with which $\mathrm{pat}h_{n}$ can categorise class *m* is $\beta_{\mathrm{nm}}\left(0\leq \beta_{\mathrm{nm}}\leq 1;,=1,2\right)$ .

Firepower of all leaf nodes belonging to a specific class is combined to determine the prediction confidence $z_{m}^{j}\;\left(m=1,2\right)$ of the jth pattern using FDT

$$y_{m}^{j}=\sum_{n=1}^{6}\mu_{\mathrm{pat}h_{n}}^{j}\times \beta_{\mathrm{nm}}$$ (13)

Predictions may be made by using the formula:

$$y_{1}^{j}=\sum_{n=1}^{6}\mu_{\mathrm{pat}h_{n}}^{j}\times \beta_{n1}y_{2}^{j}=\sum_{n=1}^{6}\mu_{\mathrm{pat}h_{n}}^{j}\times \beta_{n2}$$ (14)

Segmentation to a unique class requires selecting the classes with the greatest degree of membership, such as classifying a given pattern to categorise $m_{0}$ .

$$m_{0}=\arg\max_{m=1,2}\left\{z_{m}^{j}\right\}$$ (15)

The class with the most accurate predictions will be chosen, $m_{0}=\arg\max_{m=1,2}\left\{z_{1}^{j},z_{2}^{j}\right\}$

To fuzzify input attributes, the method selects Gaussian membership (GM) functions out of many alternatives due to its differentiable property. For jth pattern membership degree of $\mathrm{pat}h_{n}$ can be calculated by

$$\mu_{\mathrm{pat}h_{n}}^{j}=\underset{k}{∐}\mu G_{\mathrm{kn}}=\prod_{k}\exp\left(\frac{{\left(T_{k}^{j}-D_{\mathrm{kn}}\right)}^{2}}{2\sigma_{\mathrm{kn}}^{2}}\right)$$ (16)

Where the centre and standard deviation of GM of the kth input attribute on the nth route of $G_{\mathrm{kn}}$ is represented by $D_{\mathrm{kn}}$ and $\sigma_{\mathrm{kn}}$ , respectively.

The technique specifies that the FDT’s error function is a differentiable function like the mean square error *F*.

$$F=\frac{1}{2n}\sum_{m=1}^{r}\sum_{j=l}^{n}{\left(d_{m}^{j}-z_{m}^{j}\right)}^{2}$$ (17)

In SNF-DT, n is the number of training patterns, $d_{m}^{j}$ and $z_{m}^{j}$ are required classes for the jth training set.

For the error to be minimised, all variations about the parameter’s Gaussian centre locations, widths, and confidence factors must disappear. The parameter update rule is a consequence.

$$\theta^{\tau +1}=\theta^{\tau }-\eta \frac{\partial F}{\partial \theta }$$ (18)

FDT structures with Gaussian membership functions are updated using the following update rules: centers, widths, and confidence factors.

$$\beta_{\mathrm{nm}}^{\tau +1}=\beta_{\mathrm{nm}}^{\tau }+\frac{\eta }{n}\sum_{j=1}^{n}\left(e_{m}^{j}-z_{m}^{j}\right)\mu_{\mathrm{pat}h_{n}}^{2}$$ (19)

## Results

In this research, we examined the factors affecting tourist involvement in coffee tourism after the COVID-19 pandemic in Thailand. Following a thorough analysis of the sample group, which included both Thai and foreign visitors who engaged in coffee tourism in Thailand’s northern area, the following conclusions may be drawn. The parameters are satisfaction level, coffee tourism rate, prediction rate, prediction error, accuracy. The existing methods are KNN (k-nearest neighbors), ^26^ naïve Bayes, ^27^ BGVAR (Bayesian global vector auto regressive model, ^28^ ANN (artificial neural network), ^29^ SNF- DT (stochastic neuro fuzzy decision tree) [Proposed].

The survey was completed by 275 travelers who came to Thailand to visit coffee plantations. According to the replies, more than 59% of respondents overall were female, which is in line with demographic data. Between 21-30 years old, around 60% of the population. Around 58% of people held bachelor’s degrees. About 29% of respondents were students or university students, and about 75% were unmarried. 38% of those who responded said it was their first time visiting the coffee tourist locations in northern Thailand. 40% of the participants were situated in Bangkok, and 28% earned between 10,001 and 20,000 THB each month. 52% of tourists learnt about places online, and 92% supported internet advertising. 48% of tourists drove privately. 52% spent 1,000-5,000 THB for transportation. Over 75% of participants spent little more than 1,000 THB. 56% spent less than 1,000 THB for meals and drink. 53% spent less than 1,000 THB for lodging. 64% spent less than 1,000 THB on souvenirs. 68% of respondents spent no more than 1,000 THB. The majority of Thai visitors surveyed (67%), came to the places for pleasure, followed by 22% who wanted an educational experience and to sample fresh coffee from the coffee manufacturing facilities. 54% of visitors stayed 1-2 days. 77% wanted to come during coffee harvest season. 75% wanted coffee goods, especially instant coffee. Almost 47% wanted to learn about coffee tourism by visiting farms and factories. Tours to coffee estates drew 81%of participants and more than 84% of satisfied coffee tourists wanted to re-visit. Almost 90% of the examined travelers would suggest coffee tourism to others.

Second, the findings revealed demographic data, habits, requirements, and levels of satisfaction for all 125 foreign coffee tourists who responded to the questionnaire. In terms of demographic data, the results revealed that women made up the majority of the respondents, or more than 57%. Nearly 45% of the population was aged 21-30. About 33% had completed high school. 80% of them were unmarried and 41% of the population was either students or university graduates. Around 23% of the population was based in the United States of America. 44% of workers earned less than 2,000 USD per month. It was about 65% of respondents’ first time visiting a coffee tourism site. Nearly 33% of travelers said their tour guides taught them about the places. Nearly 37% reached the destination by airways. 36% of the examined foreign tourists said they have spent between 501 and 1,000 USD on transportation. 48% spent up to 100 USD on activities in the places. Almost 42% spent between 101 and 500 USD on food and drinks. 45% spent between 101 and 500 USD on lodging. Half of them spent up to 100 USD on souvenirs. Around 18% of participants spent 100 USD on souvenirs and they spent between 101 to 500 USD on other expenses.35% visited the places for recreation. Over 47% stayed one to two days. 56% wanted to come during coffee pruning season. 54% wanted local coffee-related items and handicrafts. 56% wanted to learn about coffee tourism from local guides. 54% wanted to see coffee farms and factories. In terms of satisfaction, approximately 54% of foreign coffee tourists want to return to the coffee tourism sites. Nearly 73% said they’d suggest the coffee tourism sites they visited.

**Figure 4.**
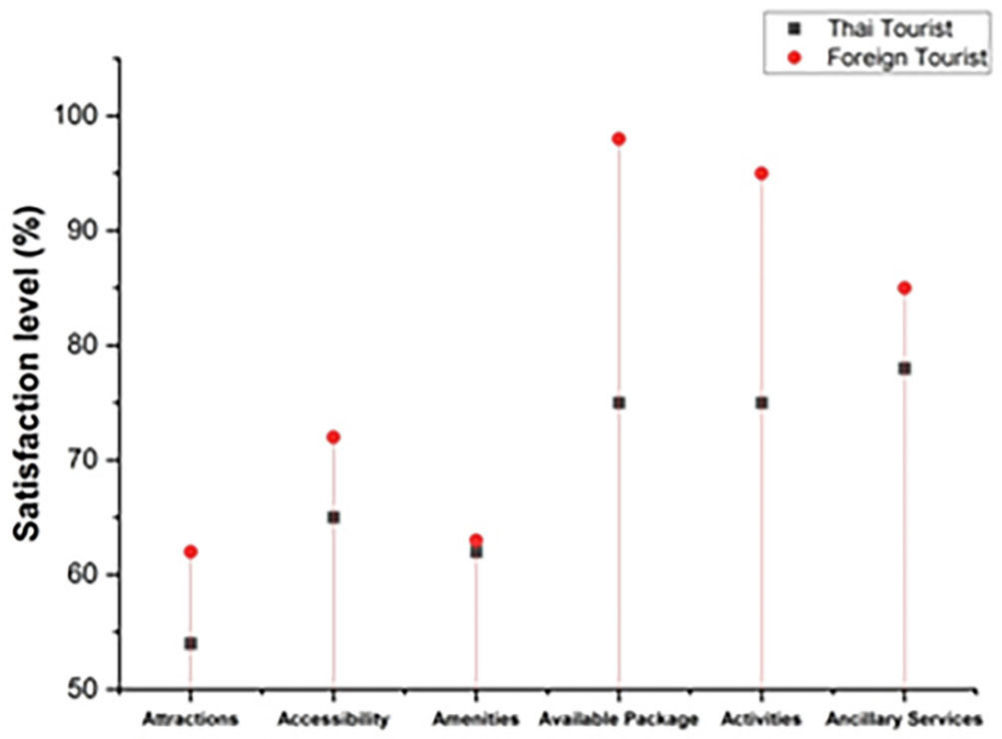
Tourists' overall satisfaction with coffee tourism in Thailand.

The degree to which an individual’s actions provide them joy and fulfillment is referred to as satisfaction. The following Figure 4 represents the satisfaction level. In this graph, it includes both Thai and foreign visitors who came to Thailand’s northern area to explore coffee tourism. The satisfaction level of visitors includes attractions, accessibility, amenities, available packages, activities, and ancillary services. The overall satisfaction level of foreign tourists is much greater than Thai tourists.

Thailand’s coffee tourism management seems to be a big hit with Thai visitors, according to the findings. Like the Thai visitors, most of the international tourists surveyed were extremely pleased with the country’s coffee tourism administration. Table 2 shows the satisfaction level of both Thai and foreign visitors.

**Table 2.** Tourist satisfaction with Thailand's coffee tourism.

Destination for coffee tourism	Foreign tourists			Thai tourists		
	SD	$\bar{x}$	Satisfaction level	SD	$\bar{x}$	Satisfaction Level
Accessibility	.654	3.81	High	.728	3.91	High
Attractions	.546	4.08	High	.623	4.13	High
Activities	.756	3.73	High	.958	3.67	High
Available package	.756	3.73	High	.990	3.66	High
Ancillary services	.785	3.28	Average	.855	3.79	High
Amenities	.623	3.79	High	.631	3.94	High

As seen by the average score of 3.8, the findings generally imply that Thai visitors are quite satisfied with the administration of the coffee tourism industry in Thailand. In a similar vein, the foreign tourists who participated in the study are generally quite satisfied with the way that coffee tourism is managed in Thailand (mean = 3.93).

**Figure 5.**
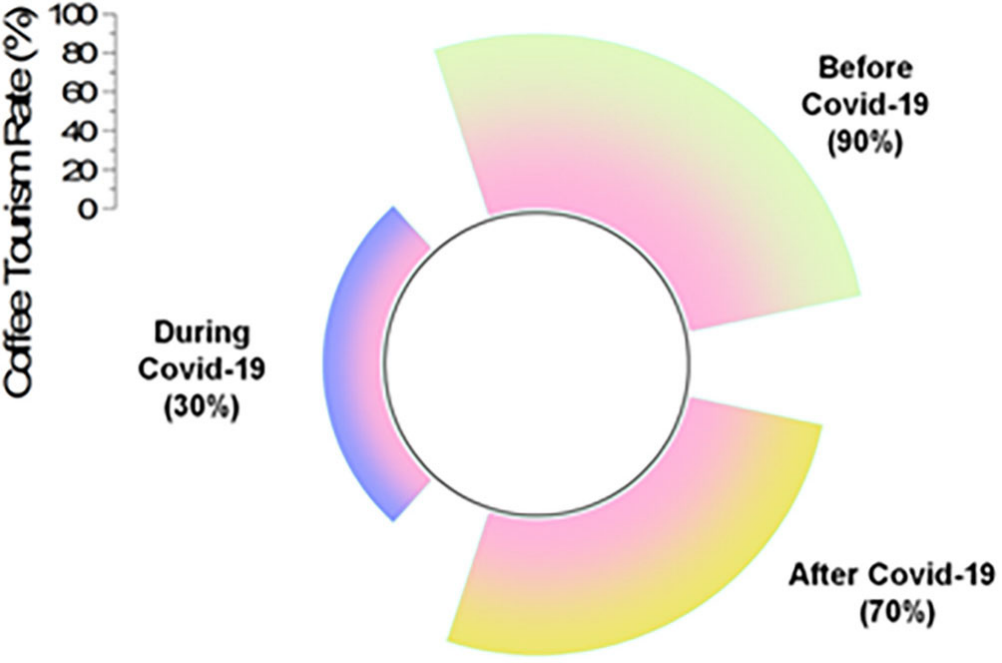
Impacts of COVID-19 in coffee tourism (source: author).

The coffee tourism industry recommends travel to coffee farms. Coffee tourism in Thailand has a lot of growth potential because of Thailand’s abundant capacity to host visitors and manage its coffee resources. The following Figure 5 represents the impacts of COVID-19 in coffee tourism.

Table 3 shows the impacts of COVID-19 on the coffee tourism rate. Table 3 illustrates that prior to COVID-19, 90% of tourists were travelling to Thailand for the purpose of coffee tourism. In the midst of the COVID-19 epidemic, this dropped to a rate of 30%, but after the pandemic, it increased to a rate of 70%.

**Table 3.** Impacts of COVID-19 in coffee tourism rate.

Coffee tourism rate		
Before COVID-19	During COVID-19	After COVID-19
90%	30%	70%

The parameters are satisfaction level, coffee tourism rate, prediction rate, prediction error, accuracy. The existing methods are KNN (k-nearest neighbors), ^26^ naïve Bayes, ^27^ BGVAR (Bayesian global vector auto regressive model, ^28^ ANN (artificial neural network), ^29^ SNF- DT (stochastic neuro fuzzy decision tree) [Proposed].

**Figure 6.**
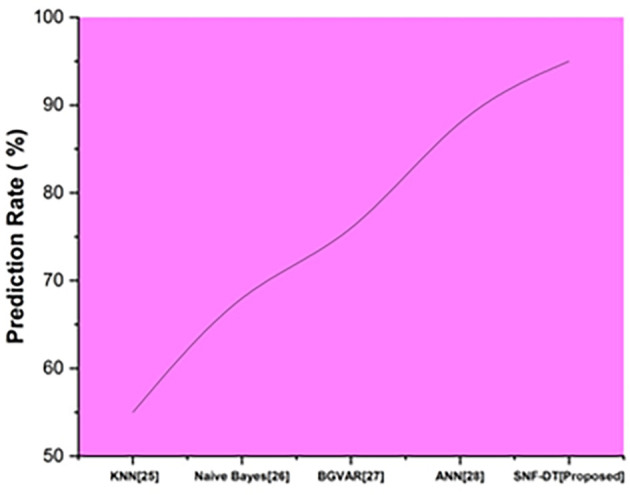
Comparative analysis of prediction rate for suggested and traditional methods.

The prediction rate is the percentage of situations in which the test findings return positive. The following Figure 6 represents the prediction rate. We evaluated the k-nearest neighbors with a prediction rate of 55%, the Naive bayes with a prediction rate of 68%, the Bayesian global vector autoregressive with a prediction rate of 76%, the artificial neural network with a prediction rate of 88%, and we proposed SNF-DT with a prediction rate of 95%. The results of the comparisons reveal that the suggested approach is superior to each of the four methods that already exist.

Table 4 shows the prediction rate for suggested and traditional methods

**Table 4.** Prediction rate for suggested and traditional methods.

Methods	Prediction rate (%)
KNN ^26^	55
Naive Bayes ^27^	68
BGVAR ^28^	76
ANN ^29^	88
SNF-DT [Proposed]	95

The following well-known equation may be used to compute the percentage prediction error:

$$\text{Percentage prediction error}=\frac{\text{measured value}-\text{predicted value}}{\text{measured value}}\times 100$$ (20)

**Figure 7.**
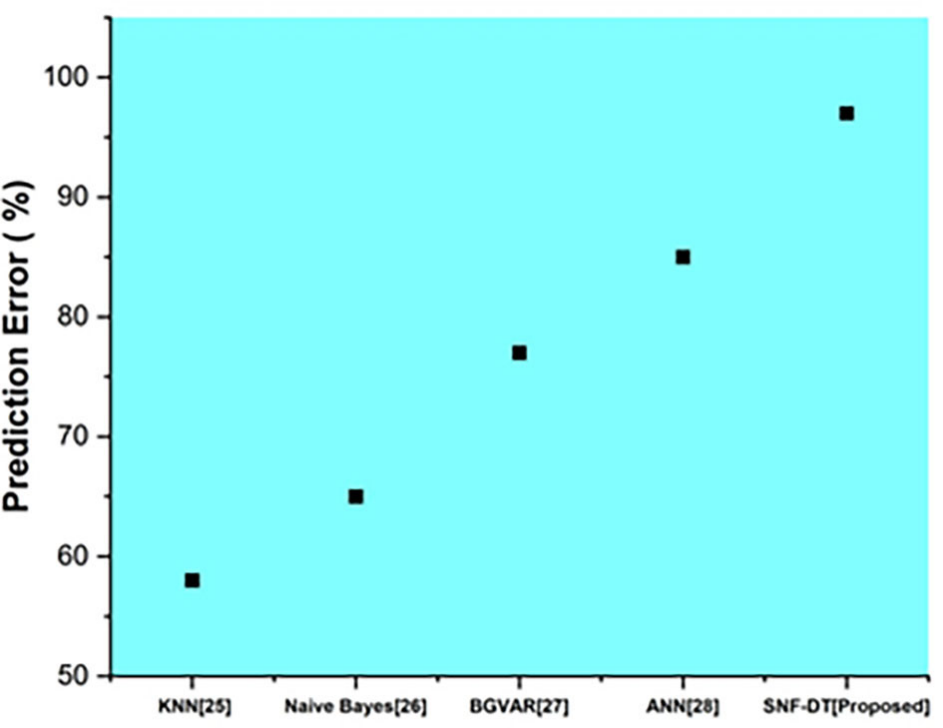
Comparative analysis of prediction error for suggested and traditional methods.

The following Figure 7 represents the prediction error. We evaluated the k-nearest neighbors with a prediction error of 58%, the Naive bayes with a prediction error of 65%, the Bayesian global vector autoregressive with a prediction error of 77%, the artificial neural network with a prediction error of 85%, and the proposed SNF-DT with a prediction error of 97%. The comparative findings show that the proposed technique is lower than the four existing approaches.

Table 5 shows the prediction error for suggested and traditional methods.

**Table 5.** Prediction error for suggested and traditional methods.

Methods	Prediction rate (%)
KNN ^26^	58
Naive Bayes ^27^	65
BGVAR ^28^	77
ANN ^29^	85
SNF-DT [Proposed]	97

The effectiveness of a classifier may be measured by counting the number of true positives (TP), true negatives (TN), false positives (FP), and false negatives (FN). ^30^ In performance evaluation, sensitivity and specificity are two metrics that are frequently utilised.

It determines how many samples are successfully categorized. It determines how exactly the outcomes correspond to the original outcome.

$$\text{Accuracy}=\frac{\mathrm{TP}+\mathrm{TN}}{\mathrm{TP}+\mathrm{TN}+\mathrm{FP}+\mathrm{FN}}$$ (21)

**Figure 8.**
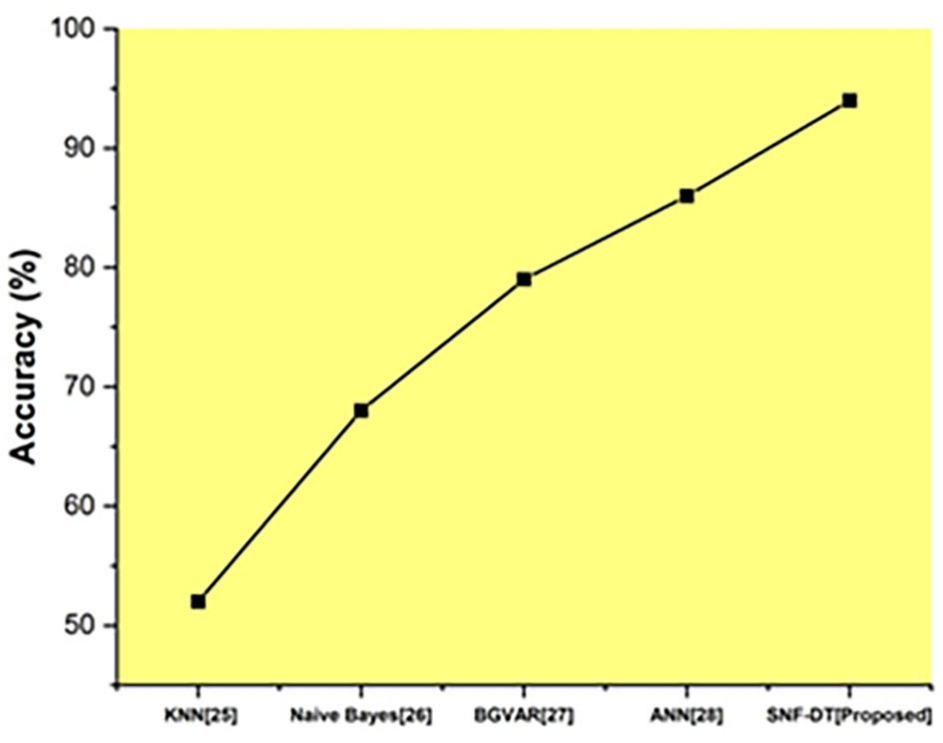
Comparative analysis of accuracy for suggested and traditional methods.

The following Figure 8 represents the accuracy. We evaluated the k-nearest neighbors with an accuracy rate of 52 percent, the Naive bayes with an accuracy rate of 68%, the Bayesian global vector autoregressive (BGVAR) with an accuracy rate of 79%, the artificial neural network with a accuracy rate of 86%, and we proposed SNF-DT with a accuracy rate of 94%. The comparative findings show that the proposed technique outperforms each of the four existing approaches.

The Table 6 shows the accuracy for suggested and traditional methods.

**Table 6.** Accuracy for suggested and traditional methods.

Methods	Prediction Rate (%)
KNN ^26^	52
Naive Bayes ^27^	68
BGVAR ^28^	79
ANN ^29^	86
SNF-DT [Proposed]	94

## Discussion

Coffee farms in Thailand were new experiences for most of the participants in the study. ^31^^-^^33^ Participants in the survey were more inclined to visit coffee enterprises in Thailand to learn about coffee tourist destinations. Coffee tourism hotspots should set up their own websites, according to the report, as a way to better connect with visitors. ^31^^,^^34^ As a result, it seems that the capacity to accommodate visitors by launching interesting coffee tourism activities that respond to known data on tourist satisfaction in this sector would likely gain their pleasure and assure return visits. ^35^^,^^36^ In this regard, it is important to understand visitor behaviour and demands so that successful marketing strategies for each province can be developed and implemented, and the necessary objectives may be met. In addition, the costs in coffee tourism sites are not exceptionally expensive since they are considered alternative tourist attractions. Inadequate knowledge about activities available in coffee tourist locations led them to arrange a one-day trip. Visitors to coffee tourism sites were more interested in having fun than in learning about the process of making and consuming coffee. The majority preferred to travel in the winter during the coffee harvest season, followed by those who wanted to visit in the rainy season for crop rotation. Consequently, most visitors to the coffee tourism attractions were located in the central area of Thailand, particularly in Bangkok. Because to the region’s lowlands, humid temperature, and short winter season, most Central Thailand’s residents spend their winters seeking a change of scenery and visiting tourist sites in other parts of the country. ^37^ Most Thai visitors want to learn more about coffee by visiting coffee estates and seeing coffee production and processing. A portion of the research team wanted to discover local culture from local tour guides. Tourists were interested in coffee farm excursions, coffee brewing demonstrations and tastings, visiting neighboring sights, seeing coffee processing facilities, and learning about local lives, cultures, and customs. Most want to return to coffee tourism hotspots and recommend them to others. ^38^ A massive dataset does not work well with KNN. ^26^ The probability outputs are not to be taken too seriously since Naive Bayes ^27^ is also recognized for being a poor estimator. BGVAR ^28^ cannot simulate distributions if it is difficult to calculate the next-symbol probability. The construction of artificial neural networks is not predetermined by any rule according to ANN. ^29^ To tackle this issue, we propose stochastic neuro-fuzzy decision trees for analyzing the coffee tourism. The findings show that coffee tourism is a popular pastime for coffee aficionados, and that many tourists enjoy a cup of joe while on vacation. Coffee- related souvenirs, tour packages, and coffee history may all be developed to improve tourism, and tourists are aware of this potential.

## Conclusion

Coffee farms have been hit hard by the current pandemic-induced change in consumer perceptions of risk and behavior. Economic, social, and health repercussions have been noticed and felt by both coffee tourists and its producers and customers alike. These methods allowed for a rapid reaction to pandemic in the coffee tourist. Data on the sample group’s visitor numbers in each province is collected and preprocessed. The linear discriminant analysis is used to extract the features, and the Bat method can be used to select the features. Stochastic neuro-fuzzy decision trees were utilized to analyze the coffee tourist data. Future research will focus on developing recommendations for coffee tourism management for local communities, adopting coffee tourism-related identities such as Robusta Coffee in the south of Thailand, and conducting a comparative study of coffee tourism administration among ASEAN and Asian countries.

## Reporting guidelines

Data are available under the terms of the Creative Commons Attribution 4.0 International license (CC-BY 4.0).

## Data Availability

figshare: Research on factors affecting tourist involvement in coffee tourism after the COVID-19 pandemic in Thailand – Questionnaire. https://doi.org/10.6084/m9.figshare.21310734.v3 This project contains the following underlying data:
- Coffee Tourism – Raw Data.xlsx figshare: Research on factors affecting tourist involvement in coffee tourism after the COVID-19 pandemic in Thailand – Questionnaire. https://doi.org/10.6084/m9.figshare.21310734.v3 This project contains the following extended data:
- Coffee Toursim - Questinnaire to Participants.docx figshare: Research on factors affecting tourist involvement in coffee tourism after COVID-19 pandemic in Thailand. https://doi.org/10.6084/m9.figshare.20417568.v4 This project contains additional extended data. Data are available under the terms of the Creative Commons Attribution 4.0 International license (CC-BY 4.0).
