# Supplementary material for: Analysis on factors affecting tourist involvement in coffee tourism after the COVID-19 pandemic in Thailand
Source: F1000Res. 2023 Nov 7;11:1269. Originally published 2022 Nov 8. [Version 3] doi: 10.12688/f1000research.123759.3 (PMC9995733; doi:10.12688/f1000research.123759.3)
Supplement: Peer review history

# Reviewer report: Reviewer response for version 2

**DOI:** 10.5256/f1000research.144118.r163419

**Published:** 2023-03-08

**Authors:** Vanessa G. B. Gowreesunkar

**Affiliations:** 1 Department of Tourism, Anant National University, Ahmedabad, Gujarat, India

I have seen the revised version. Authors have successfully addressed the comments. The paper now qualifies for indexing.

Is the work clearly and accurately presented and does it cite the current literature?

No

If applicable, is the statistical analysis and its interpretation appropriate?

Yes

Are all the source data underlying the results available to ensure full reproducibility?

No

Is the study design appropriate and is the work technically sound?

No

Are the conclusions drawn adequately supported by the results?

No

Are sufficient details of methods and analysis provided to allow replication by others?

No

Reviewer Expertise:

NA

I confirm that I have read this submission and believe that I have an appropriate level of expertise to confirm that it is of an acceptable scientific standard.

# Reviewer report: Reviewer response for version 2

**DOI:** 10.5256/f1000research.144118.r163420

**Published:** 2023-02-22

**Authors:** Ahmad R. Albattat

**Affiliations:** 1 Graduate School of Management, Post Graduate Center, Management and Science University, Selangor, Malaysia

The authors replied to my comments.

Is the work clearly and accurately presented and does it cite the current literature?

Yes

If applicable, is the statistical analysis and its interpretation appropriate?

Yes

Are all the source data underlying the results available to ensure full reproducibility?

Yes

Is the study design appropriate and is the work technically sound?

Yes

Are the conclusions drawn adequately supported by the results?

Partly

Are sufficient details of methods and analysis provided to allow replication by others?

Yes

Reviewer Expertise:

NA

I confirm that I have read this submission and believe that I have an appropriate level of expertise to confirm that it is of an acceptable scientific standard.

# Reviewer report: Reviewer response for version 1

**DOI:** 10.5256/f1000research.135896.r158786

**Published:** 2023-02-01

**Authors:** Vanessa G. B. Gowreesunkar

**Affiliations:** 1 Department of Tourism, Anant National University, Ahmedabad, Gujarat, India

This is an interesting piece and the topic of study is well justified. However, the way the paper is presented makes me doubt whether it qualifies for indexing. The work is not appropriately presented and structured as a research paper.

 The aim and objective of the study are not clearly stated. There is no research question that guides the research problem. The literature review section is mixed with the introduction section and there is no clarity on the key themes that are examined (eg coffee tourism, the profile of the case study etc). If authors claim to have used case study as their research design, they do not justify their choice. Likewise, readership might be interested to know more about Thailand. The profile of the case study is therefore missing. The methodology section is another weak section which is not appropriately presented. The work is concluded in 5 lines.

 Overall, the work lacks substance and sounds like a mish-mash of information. In my opinion, authors need to do a major revision and re-organise the whole work.

Is the work clearly and accurately presented and does it cite the current literature?

No

If applicable, is the statistical analysis and its interpretation appropriate?

Yes

Are all the source data underlying the results available to ensure full reproducibility?

No

Is the study design appropriate and is the work technically sound?

No

Are the conclusions drawn adequately supported by the results?

No

Are sufficient details of methods and analysis provided to allow replication by others?

No

Reviewer Expertise:

Tourism management, community based tourism, island tourism, marketing, communication, entrepreneurship

I confirm that I have read this submission and believe that I have an appropriate level of expertise to state that I do not consider it to be of an acceptable scientific standard, for reasons outlined above.

# Reviewer report: Reviewer response for version 1

**DOI:** 10.5256/f1000research.135896.r160144

**Published:** 2023-02-01

**Authors:** Ahmad R. Albattat

**Affiliations:** 1 Graduate School of Management, Post Graduate Center, Management and Science University, Selangor, Malaysia

1. Please remove the word "research on" from the title
2. Add the recommendation / contribution at the end of the abstract 
3. Kindly avoid using the very short sentences in the writing 
4. Must use the in-text citations in all the writing, don't leave any writing without citation
5. Re-write the problem statement and no need to highlight the title "Problem statement" just add it to the end of the introduction and followed with the research objectives 
6. You have two titles; methods and methodology, you need to use only one title to discuss your methodology part 
7. Ethics not needed here, may use it in the acknowledgment section 
8. You have mentioned that the data collection was made using the paper and pen; we are in the COVID situation, how was that possible?
9. Justify why the validation was high for the instrument, what was the sources of the instrument?
10. Add recommendation to your conclusion 
11. Update the references 

Is the work clearly and accurately presented and does it cite the current literature?

Yes

If applicable, is the statistical analysis and its interpretation appropriate?

Yes

Are all the source data underlying the results available to ensure full reproducibility?

Yes

Is the study design appropriate and is the work technically sound?

Yes

Are the conclusions drawn adequately supported by the results?

Partly

Are sufficient details of methods and analysis provided to allow replication by others?

Yes

Reviewer Expertise:

Hospitality, Events and Tourism Expert

I confirm that I have read this submission and believe that I have an appropriate level of expertise to confirm that it is of an acceptable scientific standard, however I have significant reservations, as outlined above.

# Response

**Published:** 2023-02-07

**Authors:** Warach Madhyamapurush

**Affiliations:** University of Phayao, Thailand

**Response to the Reviewer**

 Please remove the word "research on" from the title

**Answer: **Thank you for the comments. Based on the comments, the word research has been removed from the title and updated the revised title.

 Add the recommendation / contribution at the end of the abstract

**Answer: **Thank you for the comment. Based on the suggestion, the recommendation and contribution of the study has been updated in the abstract.

 "Major global businesses such as tourism have been harmed by COVID-19’s unprecedented effects. This study attempts to determine the role of coffee tourism in livelihoods based on real-time data using a machine-learning approach. More research is needed to analyze the factors of the coffee tourism experience using different machine learning approaches."

 Kindly avoid using the very short sentences in the writing 

**Answer: **Thank you for the comment. Based on the suggestion, the short sentences in the manuscript have been rewritten for flow and readability.

 Must use the in-text citations in all the writing, don't leave any writing without citation

**Answer: **Thank you for the comment. Based on the comment, entire article has been checked for the in-text citations.

 Re-write the problem statement and no need to highlight the title "Problem statement" just add it to the end of the introduction and followed with the research objectives

**Answer: **Thank you for the comment. Based on the comment, the problem statement word has been removed, and the paragraph was added after the introduction section.

 You have two titles; methods and methodology, you need to use only one title to discuss your methodology part 

**Answer: **Thank you for the comment. Based on the recommendation, I have used only “Methods” title to discuss the methodology part and updated in the manuscript.

 Ethics not needed here, may use it in the acknowledgment section 

**Answer: **Thank you for the comment. Based on the comment, the ethical section has been moved to the acknowledgement section in the revised manuscript.

 You have mentioned that the data collection was made using the paper and pen; we are in the COVID situation, how was that possible?

**Answer: **Thank you for your comments. I would like to clarify that the work was not conducted during the peak pandemic but was undertaken during the relaxation phase and after the slow influx of tourists. The data collection was made using a paper and pen, we were wearing masks and was following social distancing and other COVID safety precautionary methods. Data collection was performed using strict COVID protocols.

 Justify why the validation was high for the instrument, what was the sources of the instrument?

**Answer: **Thank you for the comments. Based on the comments, the details of validation have been updated in the manuscript.

 "This study examined the conceptual model by collecting data from a self-administered questionnaire. For the purpose of certifying its validity, the survey instrument was pretested. For the purpose of ensuring content validity, 50 national and international tourists were tested. All constructs were tested for reliability (Cronbach's alpha greater than 0.70) and survey questions were confirmed to be reliable."

 Add recommendation to your conclusion 

**Answer: **Thank you for the comment. Based on the suggestion, the recommendations have been updated in the conclusion.

 "Coffee tourism is becoming an increasingly popular travel experience, with people seeking to find the tastes of different cultures. Machine learning approaches offer the potential to explore the coffee tourism experience further. Using appropriate algorithms, researchers can gain a better understanding of consumer preferences and the experiences of those visiting coffee plantations. A predictive analytics approach can be used to analyze consumer data and identify patterns in purchasing behavior. This would allow researchers to understand better how tourists engage in coffee tourism. Additionally, sentiment analysis can be employed to analyze customer reviews and gain insight into the emotional aspects of their experiences."

 Update the references 

**Answer: **Thank you for the comments. References have been checked and updated.
